# A novel approach to evaluation of tumor response for advanced pulmonary adenocarcinoma using the intertumoral heterogeneity response score

**DOI:** 10.1002/mco2.493

**Published:** 2024-03-09

**Authors:** Xinlong Zheng, Tao Lu, Shiwen Wu, Xiaoyan Lin, Jing Bai, Xiaohui Chen, Qian Miao, Jianqun Yan, Kan Jiang, Longfeng Zhang, Xiaobing Zheng, Haibo Wang, Yiquan Xu, Weijin Xiao, Cao Li, Wenying Peng, Jianming Ding, Qiaofeng Zhong, Zihua Zou, Shanshan Yang, Yujing Li, Sihui Chen, Qiuyu Zhang, Jianfeng Yan, Guofeng Tang, Yuandong Cai, Miao kang, Tony S. K. Mok, Gen Lin

**Affiliations:** ^1^ Department of Thoracic Oncology Clinical Oncology School of Fujian Medical University, Fujian Cancer Hospital Fuzhou China; ^2^ Department of Radiology Clinical Oncology School of Fujian Medical University, Fujian Cancer Hospital Fuzhou China; ^3^ Department of Oncology Fujian Medical University Union Hospital Fuzhou China; ^4^ Department of Research Geneplus‐Beijing Institute Beijing China; ^5^ Department of Thoracic Surgery Clinical Oncology School of Fujian Medical University, Fujian Cancer Hospital Fuzhou China; ^6^ Department of Pathology Clinical Oncology School of Fujian Medical University, Fujian Cancer Hospital Fuzhou China; ^7^ The Second Department of Oncology Yunnan Cancer Hospital, The Third Affiliated Hospital of Kunming Medical University, Yunnan Cancer Center Kunming China; ^8^ Department of Radiation Oncology Clinical Oncology School of Fujian Medical University, Fujian Cancer Hospital Fuzhou China; ^9^ Institute of Immunotherapy Fujian Medical University Fuzhou China; ^10^ College of Chemistry Fuzhou University Fuzhou China; ^11^ Department of Clinical Oncology State Key Laboratory of Translational Oncology Chinese University of Hong Kong Shatin, Hong Kong Special Administrative Region China; ^12^ Fujian Key Laboratory of Advanced Technology for Cancer Screening and Early Diagnosis, Fujian Cancer Hospital Fuzhou China; ^13^ Interdisciplinary Institute for Medical Engineering Fuzhou University Fuzhou China

**Keywords:** intertumoral heterogeneity, intertumoral heterogeneous response, lung adenocarcinoma, RECIST criteria

## Abstract

Treatment response and prognosis estimation in advanced pulmonary adenocarcinoma are challenged by the significant heterogeneity of the disease. The current Response Evaluation Criteria in Solid Tumors (RECIST) criteria, despite providing a basis for solid tumor response evaluation, do not fully encompass this heterogeneity. To better represent these nuances, we introduce the intertumoral heterogeneity response score (THRscore), a measure built upon and expanding the RECIST criteria. This retrospective study included patients with 3–10 measurable advanced lung adenocarcinoma lesions who underwent first‐line chemotherapy or targeted therapy. The THRscore, derived from the coefficient of variation in size for each measurable tumor before and 4–6 weeks posttreatment, unveiled a correlation with patient outcomes. Specifically, a high THRscore was associated with shorter progression‐free survival, lower tumor response rate, and a higher tumor mutation burden. These associations were further validated in an external cohort, confirming THRscore's effectiveness in stratifying patients based on progression risk and treatment response, and enhancing the utility of RECIST in capturing complex tumor behaviors in lung adenocarcinoma. These findings affirm the promise of THRscore as an enhanced tool for tumor response assessment in advanced lung adenocarcinoma, extending the RECIST criteria's utility.

## Graphical abstract

In the clinical management of advanced pulmonary adenocarcinoma, a commonly observed phenomenon is the intertumoral heterogeneous response (THR), which refers to the significant variation in treatment responses among different tumors within the same patient. We introduce the intertumoral heterogeneity response score (THRscore), a measure built upon and expanding the RECIST criteria, to evaluate THR effectively, stratifying patients by progression risk.

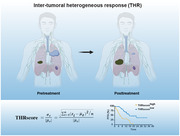

## 1 INTRODUCTION

In the clinical management of advanced pulmonary adenocarcinoma, a commonly observed phenomenon is the intertumoral heterogeneous response (THR), which refers to the significant variation in treatment responses among different tumors within the same patient.^1^, ^2^, ^3^, ^4^, ^5^ This variability in response can be attributed to a range of factors, including genetic variations among tumors that affect drug sensitivity, differences in the tumor microenvironment that impact drug delivery and efficacy, diverse immune responses within each lesion, and selective pressures induced by treatment itself that lead to the survival of drug‐resistant cells.^6^, ^7^, ^8^, ^9^ Such THR, adds considerable complexity to treatment decision‐making and prognosis estimation, posing significant challenges to oncologists.^10^, ^11^ Consequently, a thorough understanding and assessment of this heterogeneity is pivotal for developing effective treatment strategies and accurately predicting disease prognosis.

Response Evaluation Criteria in Solid Tumors (RECIST),^12^ widely adopted as the standard for assessing treatment response in solid tumors, focuses on measuring the sum of diameters of selected measurable lesions. Its modified version, RECIST 1.1,^13^ is extensively used in clinical practices and trials worldwide. However, this approach has inherent limitations in advanced pulmonary adenocarcinoma, particularly in its ability to reflect the complexities of tumor heterogeneity. RECIST's emphasis on overall tumor burden may mask the varied responses exhibited by different lesions within the same patient. Such limitations highlight a critical gap in accurately assessing the full spectrum of tumor behavior and responses, especially in cases with multiple distinct lesions. Evidence suggests that even if the RECIST criteria indicates partial response (PR) or stable disease (SD), there may still be disease progression and a shortened progression‐free survival (PFS).^14^, ^15^, ^16^ Although efforts have been made to develop improved versions such as iRECIST^17^ and PERCIST^18^ to accommodate advancements in immunotherapy and emerging imaging technologies, they still primarily focus on the overall tumor burden but neglect heterogeneity of tumor. This gap underscores the need for more comprehensive assessment tools that can capture the diverse nature of tumor response.

To bridge this existing gap, we introduce a novel approach, employing the coefficient of variation (CV)^19^ of all lesion changes as a measure of THR. The CV, the standard deviation divided by the arithmetic mean, is the most widely used measure of the extent of trait variation.^19^, ^20^ Indeed, its applications are already proven in numerous fields such as finance, meteorology, and engineering where it is effectively used to measure dispersion of data points.^21^, ^22^, ^23^ In our study, we hypothesize that by applying the CV, we can capture the THR within a patient and gain insights into the heterogeneity of treatment response.

Given this background, we aim to establish a new, clinically convenient system for assessing THR, which both builds upon and expands the existing RECIST framework. Central to this is the derivation of an intertumoral heterogeneity response score (THRscore), projected to correlate with PFS and tumor response according to RECIST 1.1. Moreover, we utilize next‐generation sequencing (NGS) to further delve into potential mechanisms that link THR with clinical outcomes. This comprehensive and integrated approach promises to enhance our understanding and management of tumor heterogeneity.

## 2 RESULTS

### 2.1 Patient characteristics

**FIGURE 1:**
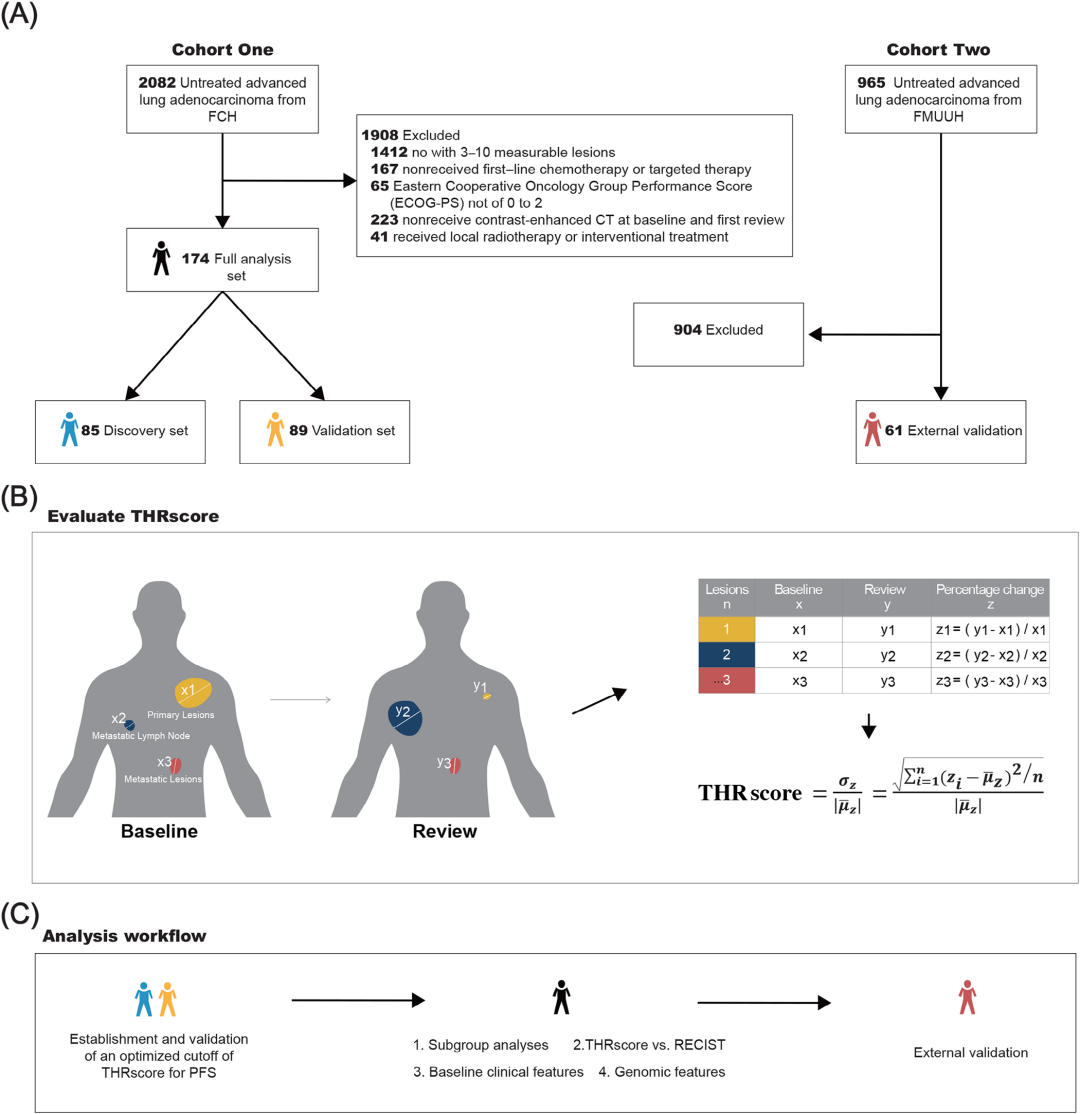
Study scheme. (A) Flowchart illustrating the data collection and sample allocation process. Cohort One comprises eligible patients assigned to either the discovery set (June 2018 to December 2020) or the validation set (January 2016 to May 2018) from Fujian Cancer Hospital (FCH). Cohort Two includes eligible patients for external validation (January 2010 to December 2020) from Fujian Medical University Union Hospital (FMUUH). (B) Schematic representation of the method used to measure the intertumoral heterogeneity response following systemic treatment. Simulating the heterogeneity of lesion changes in patients during systemic treatment: blue, enlargement; yellow, reduction; red, no change. The measurement methods for lesions and lymph nodes were conducted according to the Response Evaluation Criteria in Solid Tumors version 1.1 (RECIST) guidelines. Specifically, the maximum diameter was utilized for assessing lesions, while the shortest diameter (short axis) was employed for the evaluation of metastatic lymph nodes. (C) Main analysis workflow.

In this retrospective study, we initially screened 2082 treatment‐naïve patients in Cohort One, of which 174 met the inclusion criteria after excluding those who did not fulfill our study requirements (Figure 1A). The included patients had a median age at diagnosis of 59 years (range, 36−84 years); 105 (60%) were male and 58 (33%) were current or former smokers. Based on the time of treatment, patients were assigned to the discovery set (treated between June 2018 and December 2020) or the validation set (treated between January 2016 and May 2018). Among these included patients, 101 (58%) underwent platinum‐based doublet chemotherapy, 73 (42%) received targeted therapies, and a total of 57 (33%) patients had epidermal growth factor receptor (EGFR) mutation and received EGFR tyrosine kinase inhibitor. Significant differences were observed between the two set regarding treatment regimens, presence of brain metastases, Eastern Cooperative Oncology Group Performance Score (ECOG‐PS), number of lesions, and the presence of driver genes. Table 1 provides a detailed overview of patient characteristics.

**TABLE 1:** Baseline demographic and clinical characteristics of the patient sample.

	Full analysis set	Discovery set	Validation set	
	(*N* = 174)	(*N* = 85)	(*N* = 89)	*p*
Age (years)				
Median [range]	59 [26, 80]	59 [33, 80]	59 [26, 75]	0.597
Sex				
Female	69 (39.7%)	30 (35.3%)	39 (43.8%)	0.320
Male	105 (60.3%)	55 (64.7%)	50 (56.2%)	
Smoking				0.789
Absence	116 (66.7%)	58 (68.2%)	58 (65.2%)	
Presence	58 (33.3%)	27 (31.8%)	31 (34.8%)	
ECOG‐PS				0.029
1	162 (93.1%)	75 (88.2%)	87 (97.8%)	
2	12 (6.9%)	10 (11.8%)	2 (2.2%)	
Brain metastasis				0.017
Absence	128 (73.6%)	70 (82.4%)	58 (65.2%)	
Presence	46 (26.4%)	15 (17.6%)	31 (34.8%)	
Bone metastasis				0.181
Absence	114 (65.5%)	51 (60.0%)	63 (70.8%)	
Presence	60 (34.5%)	34 (40.0%)	26 (29.2%)	
Adrenal metastasis				0.086
Absence	149 (85.6%)	69 (81.2%)	80 (89.9%)	
Presence	25 (14.4%)	16 (18.8%)	9 (10.1%)	
Liver metastasis				0.901
Absence	149 (85.6%)	72 (84.7%)	77 (86.5%)	
Presence	25 (14.4%)	13 (15.3%)	12 (13.5%)	
Lesions number				
3	125 (71.8%)	56 (65.9%)	69 (77.5%)	0.039
4	42 (24.1%)	22 (25.9%)	20 (22.5%)	
5	4 (2.3%)	4 (4.7%)	0 (0%)	
6	3 (1.7%)	3 (3.5%)	0 (0%)	
Targetable driver genes^a^				0.008
Mutation	100 (57.5%)	59 (69.4%)	41 (46.1%)	
Wild type	60 (34.5%)	21 (24.7%)	39 (43.8%)	
Unknown	14 (8.0%)	5 (5.9%)	9 (10.1%)	
Treatment				0.007
Chemotherapy	101 (58.0%)	40 (47.1%)	61 (68.5%)	
Targeted therapy	73 (42.0%)	45 (52.9%)	28 (31.5%)	

Eligible patients were assigned to discovery set (between June 2018 and December 2020) or validation set (between January 2016 and May 2018).

Abbreviation: ECOG‐PS, Eastern Cooperative Oncology Group Performance Score.

^a^ Targetable driver genes include *EGFR*, *ALK*, *ROS1*, *RET*, *BRAF*, *MET*, and *HER2*.

### 2.2 THRscore as an independent predictor of treatment efficacy

**FIGURE 2:**
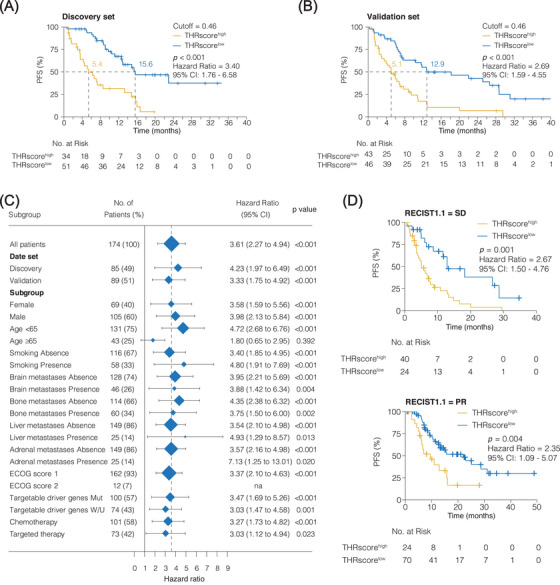
Association between intertumoral heterogeneity response score (THRscore) and prognosis. The Kaplan‐Meier curve for progression‐free survival (PFS) in the discovery set (A) and validation set (B). (C) Forest plot depicting the different stratification factors used for the PFS subgroup analyses. The horizontal line represents the 95% confidence interval (95% CI) for each group, while the vertical dotted line represents the hazard ratio (HR) for all patients. The vertical solid line represents an HR of 1. An HR above 1.0 indicates that a high THRscore is an unfavorable prognostic biomarker. Targetable driver genes include *EGFR*, *ALK*, *ROS*, *RET*, *BRAF*, *MET*, and *HER2*. (D) Kaplan–Meier curves for PFS stratified according to the THRscore for the patient with stable disease (SD) or partial response (PR), respectively. All Kaplan–Meier curves were compared using a Log‐rank statistical test. ECOG‐PS, Eastern Cooperative Oncology Group Performance Score; Mut, mutation; *n*, number of patients indicated; W/U, wild type/unknown.

In the discovery dataset (*n* = 85), the optimal cutoff for predicting PFS was determined to be 0.46 by maximally selected rank statistics (see *Methods*, Figure S1). Based on this cutoff, 51 (60%) and 34 (40%) were considered THRscore^low^ and THRscore^high^, respectively. The median PFS of the THRscore^low^ versus THRscore^high^ groups was 15.6 and 5.4 months, respectively (hazard ratios [HR], 3.40; 95% confidence interval [95% CI], 1.76−6.58; *p* < 0.001; Figure 2A). Similar findings were obtained on the validation set (*n* = 89) whereby the median PFS for the THRscore^low^ (*n* = 46) and THRscore^high^ (*n* = 43) group were 12.9 months and 5.1 months, respectively (HR, 2.69; 95% CI, 1.59−4.55; *p* < 0.001; Figure 2B).

Univariate and multivariate analyses were performed on the entire data set, and the THRscore^high^ was shown to be an independent predictive factor (HR, 2.48, 95% CI, 1.63−3.77, *p* < 0.001; Figure S2). In the subgroup analysis, the increased risk of disease progression in the patients with THRscore^high^ was apparent in almost all subgroups, including the targeted therapy group or chemotherapy group (Figure 2C). Notably, the predictive THRscore was further confirmed by stratified PR and SD analyses. Specifically, the THRscore^high^ was associated with significantly shorter PFS both in patients with SD (median PFS, 12.9 months vs. 5.1 months; HR, 2.67; 95% CI, 1.50−4.76; *p* = 0.001; Figure 2D) and with PR (median PFS, 22.0 months vs. 9.5 months; HR, 2.35; 95% CI, 1.09−5.07; *p* = 0.004; Figure 2D). The dose‐response relationship between the THRscore levels and PFS was evaluated by restricted cubic splines. While nonlinear associations were observed, higher levels of THRscore appeared to be monotonically associated with a higher risk of disease progression (*p* < 0.001; Figure S3). We divided the THRscore into quintiles. The median PFS of the quintiles were 3.9, 6.9, 14.8, 12.7, and 22.0 months, respectively. The highest versus lowest quintile HR was 5.25 (95% CI, 2.64−10.44; Table S2).

**FIGURE 3:**
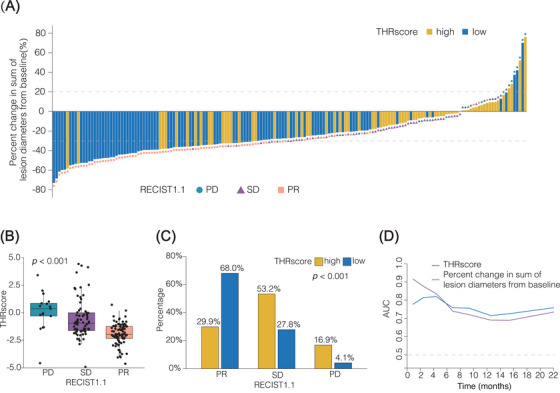
The association between intertumoral heterogeneity response score (THRscore) and clinical response in the full dataset. (A) Waterfall plot of the response of the all lesions according to RECIST for each patient. Patients were evaluable for response using RECIST1.1. (B) Boxplots illustrating the association between the THRscore and best response according to RECIST1.1. The central line on the box plot indicates the median value, the box plot limits indicate the upper and lower quartiles, and the whiskers indicate 1.5× the interquartile range. The Kruskal–Wallis test was used to evaluate the overall differences across all three groups. (C) The ratio of patients with partial response (PR), stable disease (SD), and progressive disease (PD) in the THRscore^high^ and THRscore^low^ groups. *p* < 0.001 by Fisher's exact test. (D) Time‐dependent ROC analysis for predicting disease progression. This figure presents the time‐dependent receiver‐operator characteristic (ROC) analysis, comparing the predictive accuracy of THRscore with the percent change in the sum of lesion diameters from baseline in assessing the risk of disease progression at different time points. The analysis was conducted for three times intervals: 6, 12, and 18 months. AUC, area under the ROC curve.

We have cross‐referenced THRscore with objective response rate (ORR) as per standard RECIST 1.1. Based on the full dataset, a total of 89 (51.1%) patients were defined to achieve PR, 68 (39.1%) achieved SD, and 17 (9.8%) achieved progressive disease (PD) (Figure 3A). The median THRscore of patients with PD, SD, and PR was 1.51, 0.58, and 0.27, respectively (*p* < 0.001; Figure 3B). The tumor response rate of patients with THRscore^high^ and THRscore^low^ was 30 and 68%, respectively (*p *< 0.001, Fisher's exact test; Figure 3C). In addition, Figure 3A displays the posttreatment tumor burden (sum of the diameters of all lesions), which recorded simultaneously with the evaluation of the THRscore. Further analysis using the Net Reclassification Index (NRI)^24^ and the integrated discrimination index (IDI)^24^ was conducted to compare the predictive value of THRscore with the RECIST 1.1 response at the 12‐month PFS milestone. The NRI result of 0.07 (95% CI: 0.01–0.14, *p* < 0.001) indicates a significant improvement in patient reclassification for disease progression risk when including THRscore, compared with using RECIST 1.1 alone. Additionally, the IDI result of 0.33 (95% CI: 0.04–0.42, *p* < 0.001) demonstrates THRscore's enhanced ability to discriminate between progression and nonprogression cases. These results validate the THRscore as an effective tool for assessing treatment outcomes in lung adenocarcinoma, offering more nuanced insights than traditional response criteria and highlighting its potential as a supplementary measure to RECIST 1.1.

### 2.3 Association between THRscore and clinical characteristics

**FIGURE 4:**
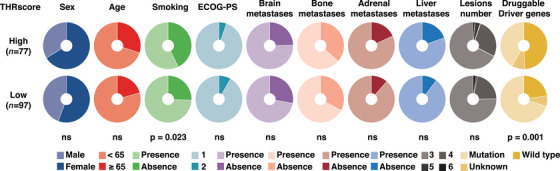
Association between intertumoral heterogeneity response score (THRscore) and clinical characteristics. Pie charts showing the distribution of different clinicopatholotgic factors in the THRscore^high^ and THRscore^low^ groups, respectively. The Fisher's exact test was used to compare the difference in the proportion between the two groups. Targetable driver genes include *EGFR*, *ALK*, *ROS*, *RET*, *BRAF*, *MET*, and *HER2*. n, number of patients indicated; ns, not statistically significant.

The patients in the THRscore^high^ group were more likely to be smokers (42.9 vs. 25.7%) and negative for targetable driver genes^25^ (including *EGFR*, *ALK*, *ROS1*, *RET*, *BRAF*, *MET*, and *HER2;* 49.4 vs. 22.6%) (*p *< 0.05, Fisher's exact test; Figure 4). There was no significant correlation between THRscore and other clinical parameters, including sex, age, ECOG‐PS, number of lesions, brain metastasis, bone metastasis, adrenal metastasis, and liver metastasis.

We also analyzed the THRscore as a continuous variable to provide a more comprehensive overview. The THRscore was a non‐normally distributed variable with a median value of 0.40 (interquartile range, 0.03−23.71) (Figures S4A and S4B). The results were generally consistent with categorical variables analyses. Specifically, patients who smoked, had liver metastasis or tested negative for the targetable driver genes had significantly higher median THRscore values (0.54 vs. 0.37, 0.57 vs. 0.38, 0.58 vs. 0.36; all *p* < 0.05; Figure S4C).

### 2.4 Genomic profiling according to THRscore

**FIGURE 5:**
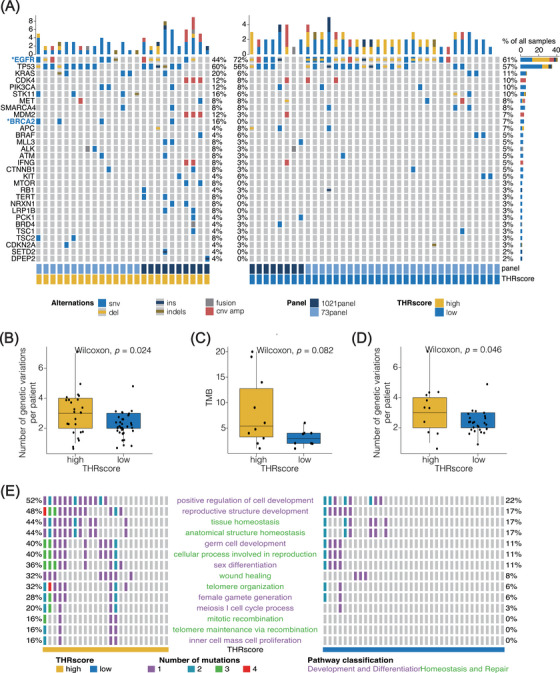
Genomic profiles and potential mechanisms linked to intertumoral heterogeneity response score (THRscore). (A) Oncoprint of mutations identified in the 61 patients. Results of targeted DNA sequencing of lung adenocarcinoma tissues. *Represents a statistically significant difference (*p* < 0.05) in the gene mutation rate between the high and low THRscore groups according to Fisher's exact test. CNV apm, copy number variation of amplification; del, deletions; SNV, single nucleotide variations; ins, small insertions. (B) Boxplots showing differences in the number of genetic variations for each patient (*n* = 61) in the high and low THRscore groups (C) Boxplots showing differences in the tumor mutation burden (TMB) in patients with high and low THRscores based on 1021‐gene panel. (D) Boxplots showing the association between the THRscore and the number of genetic variations in patients with the *EGFR* mutation (*n* = 37). (E) Waterfall plot illustrating the pathway alteration in each patient. Based on Fisher's exact test, patients with a low THRscore had significantly lower pathway alteration rates than those with a high THRscore (*p* < 0.05).

Genomic profiles based on NGS were available for a cohort of 61 patients, with 25 being THRscore^high^ and 36 being THRscore^low^. Similar to the overall population, we observed a significant difference in PFS between the two groups (HR, 3.09; CI, 1.34−7.11; *p* = 0.001, Figure S5). Figure 5A presents the genomic landscape derived from the comprehensive mutational analysis of 61 patients with lung adenocarcinoma, as conducted using Maftools.^26^ A total of 281 mutation events were identified, affecting 113 genes (Figure S6A). The types of genetic variants included single nucleotide variations (SNVs), small insertions (ins), deletions (del), indels, fusions, and copy number variations (Figure S6B). The most prevalent mutations were SNVs, which constituted a significant proportion of the alterations. Among these, the most frequently mutated genes were EGFR (found in 61% of all samples), TP53 (57%), and KRAS (11%).

*EGFR* mutation was more common in the THRscore^low^ group (72%) than in the THRscore^high^ group (44%) (*p* < 0.05; Fisher's exact test) and similarly for the BRCA2 mutation at 16 versus 0% (*p* < 0.05, Fisher's exact test; Figure 5A). The median number of mutations in the THRscore^high^ group was 3, significantly higher than in the THRscore^low^ group (Figure 5B). Correspondingly, the tumor mutation burden (TMB) of the THRscore^high^ and THRscore^low^ groups was 5.4 and 2.9, respectively (*p *= 0.08, Wilcoxon test; Figure 5C). Consistent results were also observed in patients with *EGFR* mutations (Figure 5D). No significant difference was observed in the THRscore between patients with *EGFR* mut+/*TP53* mut+ and *EGFR* mut+/*TP53* mut− group (Figure S7). Overall, patients in the THRscore^high^ group had a higher tendency for genetic mutations and a more complex genetic background.

The results of the Gene ontology (GO) enrichment analysis on the 73 genes are summarized in Table S3. THRscore^high^ group had a higher proportion of mutations in pathways related to development, differentiation, homeostasis, and repair, such as the pathways involved in the positive regulation of cell development (52 vs. 22%) and wound healing (32 vs. 8%) (Fisher's exact test, *p* < 0.05; Figure 5E and Table S4). Additionally, the patients with a high THRscore had a higher frequency of multiple genetic mutations in those pathways (Figure 5E). Conversely, mutations in pathways involved in the developmental processes, such as gliogenesis and morphogenesis of an epithelial fold, were associated with a low THRscore (Table S4).

### 2.5 External validation of THRscore for predicting treatment efficacy

**FIGURE 6:**
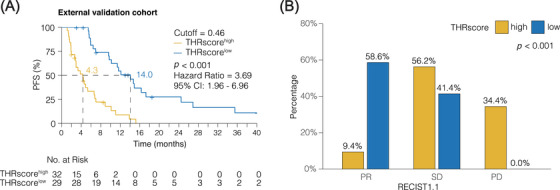
External validation of intertumoral heterogeneity response score (THRscore) for predicting treatment efficacy. (A) The Kaplan–Meier curve for progression‐free survival (PFS) in the external validation cohort. (B) The ratio of patients with partial response (PR), stable disease (SD), and progressive disease (PD) in the THRscore^high^ and THRscore^low^ groups. *p* < 0.001 by Fisher's exact test.

To further validate the utility of THRscore in a broader clinical context, we expanded our analysis to include an external cohort, reviewing 965 untreated advanced lung adenocarcinoma cases, of which 61 met our study criteria. These patients, predominantly treated with chemotherapy (*n* = 38, 62%) and targeted therapy (*n* = 23, 38%), are detailed in Table S5. Utilizing the optimal cutoff (0.46) from our discovery cohort, significant results emerged in the external validation set. The median PFS for the THRscore^high^ group (*n* = 32) was 4.3 months, compared with 14.0 months in the THRscore^low^ group (*n* = 29) (HR, 3.69; 95% CI, 1.96−6.96; *p* < 0.001; Figure 6A). Furthermore, the tumor response rate was markedly different between the two groups, with 30% in the THRscore^high^ and 68% in the THRscore^low^ categories (*p* < 0.001, Fisher's exact test; Figure 6B), underlining the predictive value of THRscore in assessing treatment outcomes in lung adenocarcinoma. In predicting 12‐month PFS, NRI and IDI analyses showed values of 0.18 (95% CI: 0.02−0.30, *p* < 0.001) and 0.33 (95% CI: 0.01−0.73, *p* < 0.001), respectively. These results demonstrate that THRscore enhances patient risk reclassification and progression risk differentiation more effectively than traditional response criteria at the 12‐month mark.

## 3 DISCUSSION

In this study, we introduced THRscore, a novel approach designed to address intertumoral heterogeneity in response to systemic therapy in advanced pulmonary adenocarcinoma. By establishing a cutoff value of 0.46, validated across cohorts, we demonstrate that THRscore effectively predicts PFS, offering a significant enhancement over the standard RECIST criteria. This innovation lies in THRscore's capacity to capture the variability in tumor responses across lesions, a critical aspect often overlooked by RECIST. Thus, THRscore serves as a potential supplement to RECIST, providing a more detailed and personalized assessment of treatment efficacy in complex cancer cases.

RECIST is an internationally recognized standardized framework for assessing tumor response and is commonly used in clinical trials for the determination of treatment response and disease progression. Previous research indicates a probable link between response classification and PFS.^27^, ^28^ In our study and external validation from another center, with correlation coefficients of 0.32 and 0.21 respectively, suggest a moderate to weak positive association between absolute ORR and PFS. However, other studies (*n* = 12) across 10 cancer types revealed a wide range of correlation coefficients between absolute ORR and PFS, ranging from −0.72 to 0.96.^29^, ^30^, ^31^ Tumor heterogeneity is a key contributing factor to these observations, with some lesions responding well to treatment while others do not. The nonresponding lesions are the ones that lead to overall disease progression.^32^, ^33^ However, the current RECIST is based on the summation of the maximum diameters of measurable lesions and does not take into account tumor heterogeneity. By adding the CV of changes in the maximum diameter of each measurable lesion, we may reflect tumor status more comprehensively. Furthermore, within the same RECIST response category, the THRscore can effectively identify individuals at high risk of disease progression. Therefore, our study may improve the current RECIST guidelines by taking tumor heterogeneity quantitatively into account.

In exploring the factors contributing to a higher THRscore in patients with advanced pulmonary adenocarcinoma, our study identified several key aspects. We observed that patients without driver oncogenes, who typically undergo chemotherapy, were more likely to have a high THRscore. This trend might be reflective of the cancer branching evolution, a prevalent pathway from the state of primary chemotherapy‐naïve to advanced chemotherapy‐treated stages.^34^, ^35^ Such a progression underscores the complex nature of tumor evolution under therapeutic pressure. Furthermore, our findings indicate that smoking is another significant factor associated with higher THRscore. The correlation between smoking and increased THRscore may be due to the cumulative mutagenic effects of tobacco carcinogens, which could drive genomic complexity and tumor heterogeneity in smokers.^36^, ^37^ The THRscore, in this context, emerges as a vital indicator of underlying tumor heterogeneity and molecular characteristics that influence treatment outcomes. A higher THRscore, indicative of a more complex genomic profile and greater mutation burden, suggests a broader spectrum of tumor behavior and response to therapy. Understanding these correlations and their implications is critical for tailoring effective treatment strategies and accurately predicting disease prognosis in patients with advanced pulmonary adenocarcinoma.

Genomic information obtained through next‐generation sequencing (NGS) provides insight into the underlying mechanisms accounting for the differentiation in THRscore. Clonal diversity is an important predictor of tumor drug‐resistance and disease progression.^38^, ^39^ Patients with higher THRscore were confirmed to have a more complex genomic profile and a higher mutation burden. This is corroborated by the report of Brady et al.^40^ which drew a correlation between higher mutation burden and inter‐and intratumor heterogeneity in prostate cancer. Interestingly, our study showed that 16% of THRscore^high^ patients had *BRCA2* mutations, while none of the patients in the THRscore^low^ group had the mutation. *BRCA2* is instrumental in the DNA damage response (DDR) pathway, and its mutation can lead to abrogation of DDR, thereby driving genomic instability and tumor heterogeneity.^41^, ^42^, ^43^, ^44^ This insight aligns with previous studies indicating that genetic mutations in key DDR players often result in genomic instability and promote tumor heterogeneity.^44^, ^45^, ^46^, ^47^, ^48^, ^49^, ^50^ Moreover, these genetic alterations can impinge upon the tumor cells' ability to undergo normal apoptosis and repair, potentially exacerbating genomic instability and tumor heterogeneity, and leading to increased resistance to treatment.^51^ This complex genomic landscape associated with higher THRscore underscores the possible association between the diverse mutation profiles and their impact on therapeutic outcomes. In this context, we also observed a considerable enrichment of alterations in pathways related to development, differentiation, homeostasis, and repair in the THRscore^high^ group. The implications of these altered pathways in the context of disease progression and treatment resistance warrant further investigation.

This study is limited by the retrospective nature of study design and the relatively small sample size. The THRscore system may also not applicable to nonmeasurable disease such as pleural effusion and bone metastasis. In addition, the reported tumor size as per imaging by computed tomography (CT) scans is subjected to observer variations thus we have adopted central evaluation of these images. Finally, the lack of data from patients treated with first‐line immunotherapy challenges the generalization of this study. Therefore, further prospective study is warranted to validate the efficacy of the THRscore in this group of patients.

In conclusion, THRscore is a simple and clinically applicable tool that may address heterogenous response to systemic therapy in advanced lung cancer. This novel tool may supplement RECIST for predicting clinical outcomes and help clinicians refine treatment strategies.

## 4 MATERIALS AND METHODS

### 4.1 Patients

We reviewed the medical records of all treatment‐naïve patients who presented with advanced stage lung adenocarcinoma at Fujian Cancer Hospital (FCH, Cohort One) between January 2016 and December 2020 were reviewed. The inclusion criteria were as follows: (i) received first‐line platinum‐based chemotherapy or targeted therapy, (ii) ECOG‐PS of 0–2, (iii) had 3–10 measurable lesions, and (iv) CT assessments being available for all measurable lesions during regular follow‐up until disease progressed. Patients who received local radiotherapy or interventional treatment during first‐line treatment were excluded (Figure 1A). Eligible patients were assigned to discovery set (between June 2018 and December 2020) or validation set (between January 2016 and May 2018).

In addition, we included an external validation cohort of consecutive cases from Fujian Medical University Union Hospital (FMUUH, Cohort Two), covering the period from January 2010 to December 2020. The same inclusion criteria were applied to this external cohort, ensuring a consistent standard across both centers.

### 4.2 Response assessment

Baseline tumor assessments were performed within two weeks prior to first line treatment, and follow‐up assessments were conducted every 4−6 weeks for 6 months, and subsequently every 12 weeks until objective disease progression, initiation of alternative therapy, or death. Tumor response was evaluated by independent radiologists adopting the RECIST 1.1.^13^ The ORR was defined as the percentage of patients with confirmed complete response or PR. PFS was defined as the time from the beginning of treatment to the date of PD or death from any cause. Patients who had not progressed were censored at the date of their last scan.

### 4.3 THRscore calculation

The THRscore is a modified version of the CV^52^ for all baseline measurable lesions included in the RECIST evaluation. The score is derived from the numerical changes in lesions after treatment, encompassing both increases (positive values) and decreases (negative values). To ensure a meaningful and non‐negative result, the CV is calculated using the absolute value of the mean.^52^, ^53^, ^54^ The THRscore evaluation is performed at 4−6 weeks after cycle 1 of first line treatment. The calculation procedure is illustrated in Figure 1B and formula is as follows:

$$\mathrm{THRscore}\;=\frac{\sigma_{z}}{\left|{\bar{\mu }}_{z}\right|}\;\;=\frac{\sqrt{\sum_{i=1}^{n}{\left(z_{i}-{\bar{\mu }}_{z}\right)}^{2}/n}}{\left|{\bar{\mu }}_{z}\right|}$$

where “*n*” refers to the number of lesions, and “z” indicates the percentage diameter change of each measurable lesion taken as the reference baseline diameter. The THRscore is defined as the ratio of the standard deviation (σ) to the absolute value of the mean of “z” ($\bar{\mu }$).

### 4.4 Establishment of cutoff THRscore

We used the maximally selected rank statistics^55^ to determine the optimal cutoff value for the THRscore, and ensured its reliability in predicting PFS through repeated sampling. Specifically, we randomly selected 70% of observations from the discovery set, and the classification system was based on an optimized threshold obtained through maximally selected rank statistics from the “*maxstat*” R package, with the minimum proportion of observations per group set at 30%. This process was iterated 10,000 times, and the final optimal cutoff value was calculated based on the highest probability from the 10,000 thresholds (see results). Our analysis workflow is presented in Figure 1C.

### 4.5 Next‐generation sequencing

The methods for preparing DNA and sequencing libraries were previously described.^56^ A panel of 73 or 1021 cancer‐related genes was used for the DNA sequencing (Table S1). Libraries were sequenced to a uniform median coverage of 515×. Somatic mutations were identified by locating the variant allele fractions above 2% with at least five high‐quality reads (Phred score ≥ 30, mapping quality ≥ 30, and without paired‐end read bias). The TMB was calculated as the number of all the nonsynonymous mutations per 0.7 Mb of the targeted coding region.

### 4.6 GO enrichment analysis

GO^57^ is a standardized system of describing genes and their functions, commonly used to understand biological processes and complex signaling pathways. Enrichment analysis and pathway analysis were performed using the “*clusterProfiler*” R package (v4.0.0) with Benjamini–Hochberg adjusted *p* value (*p*.adjust) below 0.05.

### 4.7 Statistical analysis

The primary endpoint of this retrospective study was evaluating the THRscore's predictive ability for PFS in advanced pulmonary adenocarcinoma. Given the retrospective nature of the study, a post‐hoc power analysis was conducted to assess the statistical power based on the observed data and effect size.

Categorical variables were summarized as frequencies and percentages, and continuous variables were summarized as medians and ranges. Categorical variables were compared by chi‐square analysis or Fisher exact test, while the continuous variables were compared by the Wilcoxon test or Kruskal–Wallis test as appropriate. Cox regression was used to estimate the HR for each variable at the 95% CI adjusted for potential confounders. PFS was plotted using the Kaplan–Meier method. The dose‐response relationship was examined with 4‐knot restricted cubic splines.^58^ Multivariate Cox regression was used to determine whether the THRscore remained an independent predictor of PFS after adjusting for clinical variables. The significant variables in the univariable analyses were included in the multivariable analysis. The NRI and the IDI were calculated using the *“survIDINRI”* package in R,^59^, ^60^ further assessing the predictive value of the THRscore. All data were analyzed using the R software, version 4.1.0, with RStudio, version 1.4.1717 (R Foundation for Statistical Computing). For all statistical tests, a *p* value below 0.05 was considered statistically significant.

## AUTHOR CONTRIBUTIONS

*Conceptualization*: T. M., G. L., and X. Z. *Methodology*: T. M. and G. L. *Investigation*: Q. M., K. J., L. Z., X. Z., H. W., Y. X., W. X., C. L., W. P., J. D., Q. Z., Z. Z., S. Y., Y. L., S. C., J. Y., G. T., Y. C., K. M., X. L., and J. Y. *Writing—original draft*: X. Z., T. L., and S. W. *Writing—review and editing*: T. M., J. B., X. Z., X. C., and G. L. All authors have read and approved the final manuscript.

## CONFLICT OF INTEREST STATEMENT

Jing Bai is employed by Geneplus‐Beijing Institute. There is no financial support releated to the work discussed. The other authors declare that they have no conflict of interest.

## ETHICS STATEMENT

This research was conducted in strict adherence to the ethical guidelines set by the relevant institutional and national committees overseeing human experimentation, aligning with the principles of the 1975 Helsinki Declaration and its subsequent amendments in 2000. The Fujian Cancer Hospital's Ethical Committee granted approval for this study (No. K2023‐135‐01), with a waiver for informed consent. Meanwhile, the institutional review boards of the other participating sites waived the ethics requirement, given the role of the research assistants in the study.

## Supporting information

Supporting Information

Supporting Information

## Data Availability

All data supporting the findings of this study are included in the main text or the supplementary materials. NGS data and code used to replicate our analysis are available upon reasonable request from the author, Xinlong Zheng, who can be contacted at zhengxinlong629@gmail.com.
