# Supplementary material for: A novel approach to evaluation of tumor response for advanced pulmonary adenocarcinoma using the intertumoral heterogeneity response score
Source: MedComm (2020). 2024 Mar 9;5(3):e493. doi: 10.1002/mco2.493 (PMC10924640; doi:10.1002/mco2.493)
Supplement: Supplementary file 1 — Supporting Information [file MCO2-5-e493-s001.pdf]

Supplementary Materials for

A Novel Approach to Evaluation of Tumor Response for Advanced Pulmonary Adenocarcinoma using the Inter-tumoral Heterogeneity Response Score (THRscore)

Xinlong Zheng, Tao Lu, Shiwen Wu *et al.*

*Corresponding author. Email: fjzllg133@fjzlhospital.com

This PDF file includes:

Figures S1 to S7
Tables S1 to S5

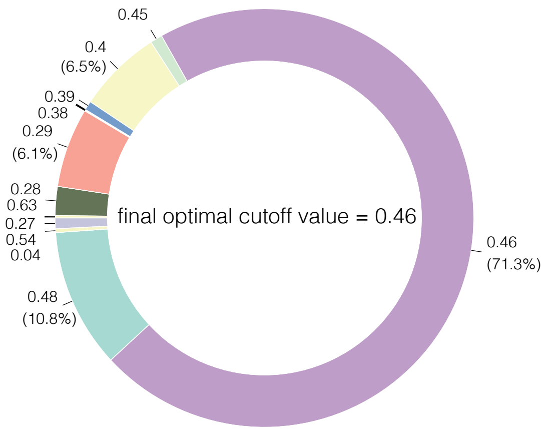

Figure S1. Stability Analysis of Optimal Cutoff Value Distribution: Results from 10,000 Random Samples. Pie chart showing the distribution of optimal cutoff values obtained from 10,000 random samplings, each time selecting 70% of the samples from the discovery group and calculating the optimal cutoff value using maximally selected rank statistics. The optimal cutoff value of 0.46 accounted for the largest proportion at 71.3%, emphasizing its robustness as the optimal threshold.

	HR	lower 95%CI	upper 95%CI	P value
Univariate Analysis				
Sex (Male vs. Female)	1.33	0.90	1.95	0.150
Age (>=65 vs. <65)	1.28	0.84	1.97	0.250
Smoking (P vs. A)	1.79	1.20	2.65	0.004
ECOG score (1 vs. 2)	2.52	0.80	7.94	0.115
Bone metastasis (P vs. A)	0.80	0.53	1.20	0.276
Brain metastasis (P vs. A)	1.07	0.70	1.63	0.747
Adrenal metastasis (P vs. A)	1.26	0.73	2.18	0.406
Liver metastasis (P vs. A)	2.24	1.40	3.59	0.001
Targetable driver genes (Wild/Unknown vs. Mut)	2.58	1.76	3.78	<0.001
Treatment (Chemotherapy vs. Targeted therapy)	2.70	1.79	4.06	<0.001
THRscore (high vs. low)	3.35	2.27	4.94	<0.001
Multivariate analysis				
Smoking (P vs. A)	1.37	0.92	2.06	0.123
Liver metastasis (P vs. A)	2.08	1.28	3.36	0.003
Targetable driver gene (Wild/Unknown vs. Mut)	1.40	0.80	2.43	0.234
Treatment (Chemotherapy vs. Targeted therapy)	1.60	0.87	2.93	0.130
THRscore (high vs. low)	2.48	1.63	3.77	<0.001

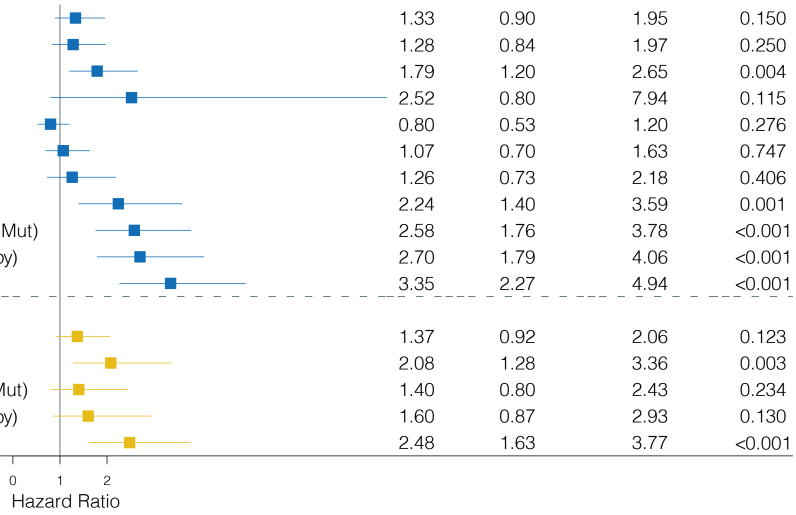

Figure S2. Identifying Independent Prognostic Factors for Progression-Free Survival in Patients with Lung Adenocarcinoma using Univariate and Multivariate Cox Regression Analyses. Univariate and multivariate analyses were performed on the entire data set, and the THRscorehigh was shown to be an independent predictive factor. Multivariable Cox regression analysis of significant covariates from univariable analyses. Targetable driver genes include EGFR, ALK, ROS1, RET, BRAF, MET, and HER2.

Abbreviations: A, Absence; P, Presence; THRscore, Tumor response heterogeneity score; ECOG, Eastern Cooperative Oncology Group; Wild, Wild type; Mut, Mutation.

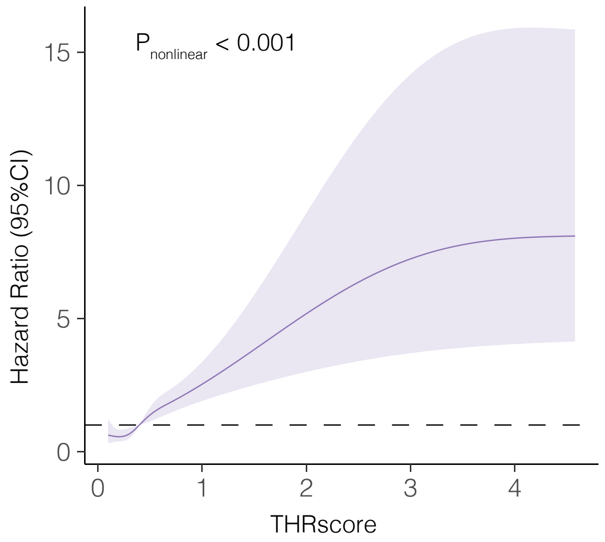

Figure S3. Restricted Cubic Spline of the Association between Inter-tumoral Heterogeneity Response Score (THRscore) and the Risk of Disease Progression. The curve is modeled using restricted cubic spline function with 4 knots. Dotted line indicates Hazard Ratio = 1.

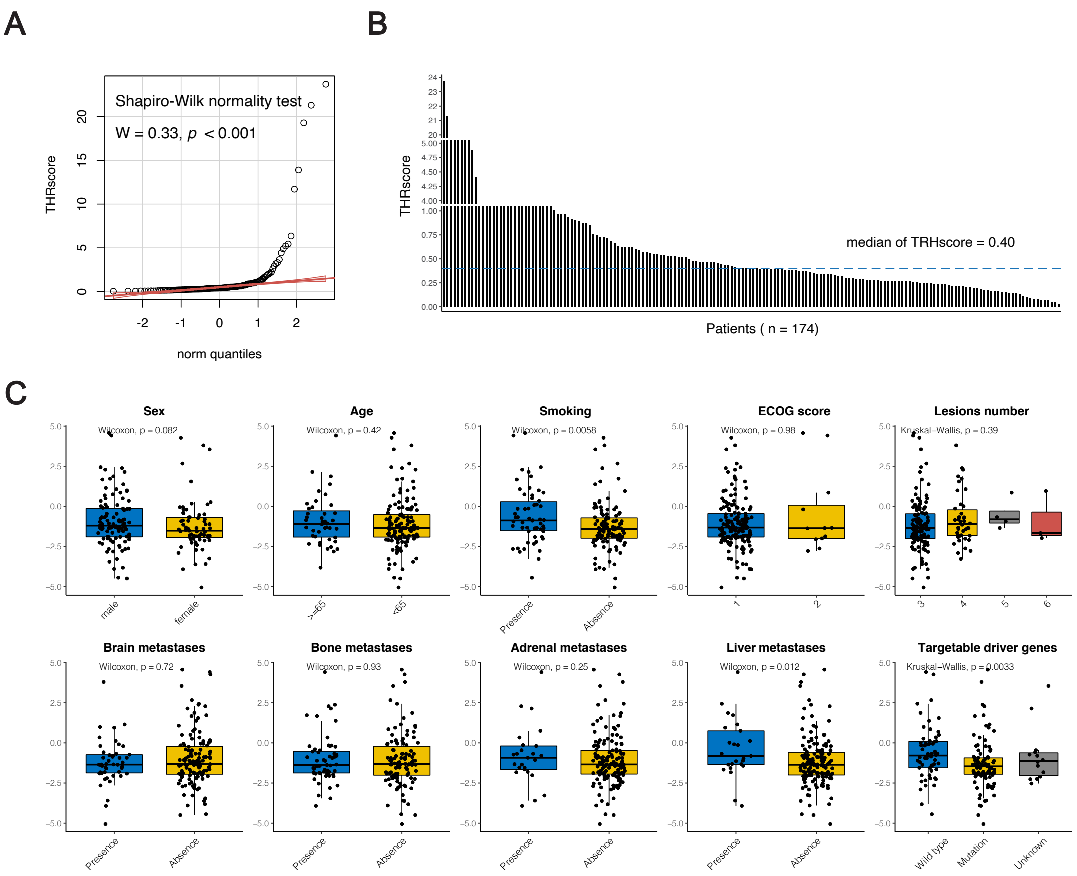

Figure S4. Association Between Inter-tumoral Heterogeneity Response Score (THRscore) and Clinical and Pathological Features. (A) Quantile-quantile (QQ) plots for the THRscore. The Shapiro–Wilk normality test indicated that the THRscore was not normally distributed ($p < 0.001$). (B) Bar graph showing the distribution of the THRscore, from left (large values) to right (small values). Each bar represents the THRscore for one of the patients. The dashed line represents the median THRscore. (C) Boxplot showing the association between THRscore and the clinical and pathological features. The central line on the box plot indicates the median value, the box plot limits indicate the upper and lower quartiles, and the whiskers indicate 1.5× the interquartile range. The Wilcoxon rank-sums test was used to evaluate the specific inter-group differences between two groups, and the Kruskal–Wallis test was used to assess the overall differences between three or four groups.Targetable driver genes include *EGFR*, *ALK*, *ROS*, *RET*, *BRAF*, *MET*, and *HER2*.

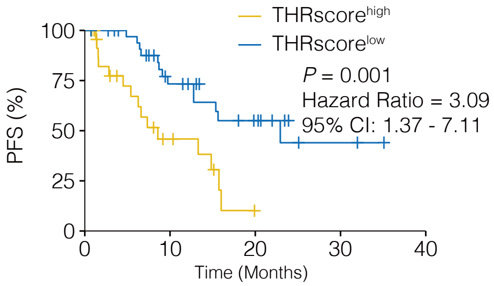

No. at Risk

THRscore^{high}	25	7	0	0	0
THRscore^{low}	36	20	8	3	0

Figure S5: Kaplan-Meier Survival Curve Analysis of Inter-tumoral Heterogeneity Response Score (THRscore) in Patients with Next-Generation Sequencing Results. Genomic profiles based on next-generation sequencing were available for a cohort of 61 patients, with 25 being THRscorehigh and 36 being THRscorelow. Kaplan-Meier curves were compared using a Log-rank statistical test.

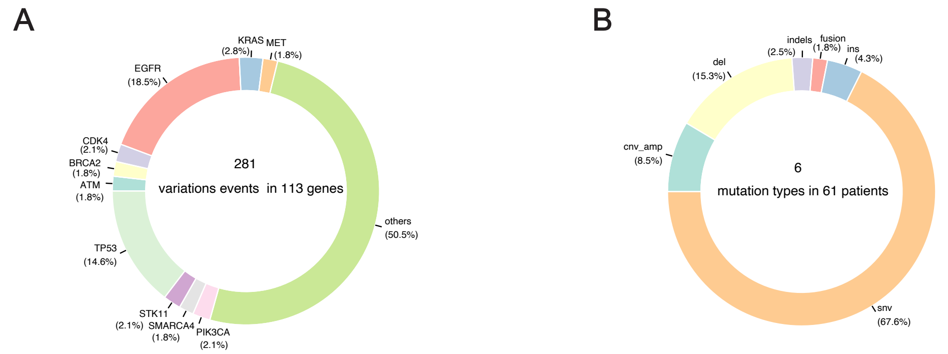

Figure S6: The Genomic Landscape in the 61 Patients. (A) Summary statistics showing gene variation events; a total of 281 mutation events occurred in 113 genes. (B) Summary statistics showing types of genetic variants; a total of 6 variant types were found in 61 patients.

Abbreviations: single nucleotide variations (SNV), small insertions (ins), deletions (del), copy number variation of amplification (CNV amp).

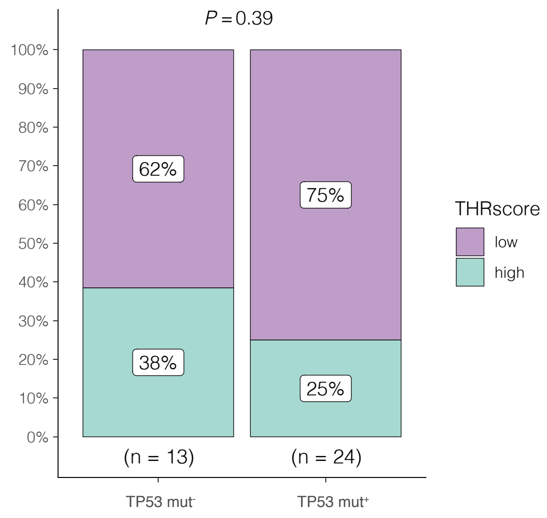

Figure S7: Inter-tumoral Heterogeneity Response Score (THRscore) Comparison between *EGFR* mut+/*TP53* mut+ and *EGFR* mut+/*TP53* mut- Groups. Bar chart displays the results of the Fisher's exact test on *TP53* mutation status factors for THRscore among *EGFR* mutation-positive patients.

Table S1. Genes List (provided as Excel file).

Table S2. Progression-Free Survival Hazard Ratios by Quintile of Inter-tumoral Heterogeneity Response Score (THRscore) in Full Dataset

				Univariable Analysis			Multivariable Analysis [*]		
**Quintile of THRscore Levels**	**No. of Patients**	**Median**	**Quintile Value**	**HR**	**95% CI**	**Trend *P***	**HR**	**95% CI**	**Trend *P***
**1(ref)**	35	0.16	< 0.24	1		**< 0.001**	1		**< 0.001**
**2**	35	0.28	0.24–0.35	1.09	0.56–2.10		1.4	0.69–2.86	
**3**	34	0.4	0.35–0.49	1.04	0.52–2.06		0.85	0.42–1.71	
**4**	35	0.62	0.49–0.95	3.26	1.78–6.00		2.44	1.22–4.9	
**5**	35	1.99	≥0.95	6.49	3.49–12.05		5.25	2.64–10.44	

[*] Adjusted for age, sex, smoking, ECOG score, brain metastases, adrenal metastases, liver metastases, and bone metastases, lesions number, targetable driver genes, and treatment in multivariable analysis.
Abbreviations: HR, hazard ratio; CI, confidence interval.

Table S3. 908 Significantly Enriched GO Terms on 73 Genes (provided as Excel file).

Table S4. Analysis of Underlying Mechanisms Associated with Inter-tumoral Heterogeneity Response Score (THRscore) (provided as Excel file)

Table S5. Clinical Characteristics of Patients in the External Validation Cohort

	**External Validation Cohort** (N=61)
**Age (years)**	
Median [Range]	60 [39, 85]
**Sex**	
Female	24 (39.3%)
Male	37 (60.7%)
**ECOG score**	
1	52 (85.2%)
2	9 (14.8%)
**Brain Metastasis**	
Absence	49 (80.3%)
Presence	12 (19.7%)
**Bone Metastasis**	
Absence	50 (82.0%)
Presence	11 (18.0%)
**Adrenal Metastasis**	
Absence	58 (95.1%)
Presence	3 (4.9%)
**Liver Metastasis**	
Absence	55 (90.2%)
Presence	6 (9.8%)
**Lesions number**	
3	34 (55.7%)
4	17 (27.9%)
5	7 (11.5%)
6	1 (1.6%)
7	1 (1.6%)
10	1 (1.6%)
**Treatment**	
Chemotherapy	38 (62.3%)
Targeted therapy	23 (37.7%)

Abbreviations: ECOG, Eastern Cooperative Oncology Group.
